# Emerging Trends in Artificial Intelligence-Assisted Colorimetric Biosensors for Pathogen Diagnostics

**DOI:** 10.3390/s26020439

**Published:** 2026-01-09

**Authors:** Muniyandi Maruthupandi, Nae Yoon Lee

**Affiliations:** 1 Department of BioNano Convergence, Gachon University, 1342 Seongnam-daero, Sujeong-gu, Seongnam-si 13120, Gyeonggi-do, Republic of Korea; maruthu1328@gachon.ac.kr; 2 Department of BioNano Technology, Gachon University, 1342 Seongnam-daero, Sujeong-gu, Seongnam-si 13120, Gyeonggi-do, Republic of Korea

**Keywords:** artificial intelligence, machine learning, deep learning, biosensor, colorimetric biosensor, bacterial pathogen, viral pathogen, diagnostics, infectious diseases, point-of-care testing

## Abstract

Infectious diseases caused by bacterial and viral pathogens remain a major global threat, particularly in areas with limited diagnostic resources. Conventional optical techniques are time-consuming, prone to operator errors, and require sophisticated instruments. Colorimetric biosensors, which convert biorecognitive processes into visible color changes, enable simple and low-cost point-of-care testing. Artificial intelligence (AI) enhances decision-making by enabling learning, training, and pattern recognition. Machine learning (ML) and deep learning (DL) improve diagnostic accuracy, but they do not autonomously adapt and are pre-trained on complex color variation, whereas traditional computer-based methods lack analysis ability. This review summarizes major pathogens in terms of their types, toxicity, and infection-related mortality, while highlighting research gaps between conventional optical biosensors and emerging AI-assisted colorimetric approaches. Recent advances in AI models, such as ML and DL algorithms, are discussed with a focus on their applications to clinical samples over the past five years. Finally, we propose a prospective direction for developing robust, explainable, and smartphone-compatible AI-assisted assays to support rapid, accurate, and user-friendly pathogen detection for health and clinical applications. This review provides a comprehensive overview of the AI models available to assist physicians and researchers in selecting the most effective method for pathogen detection.

## 1. Introduction

### 1.1. Sensing Challenges in Pathogen Detection

**Figure 1:**
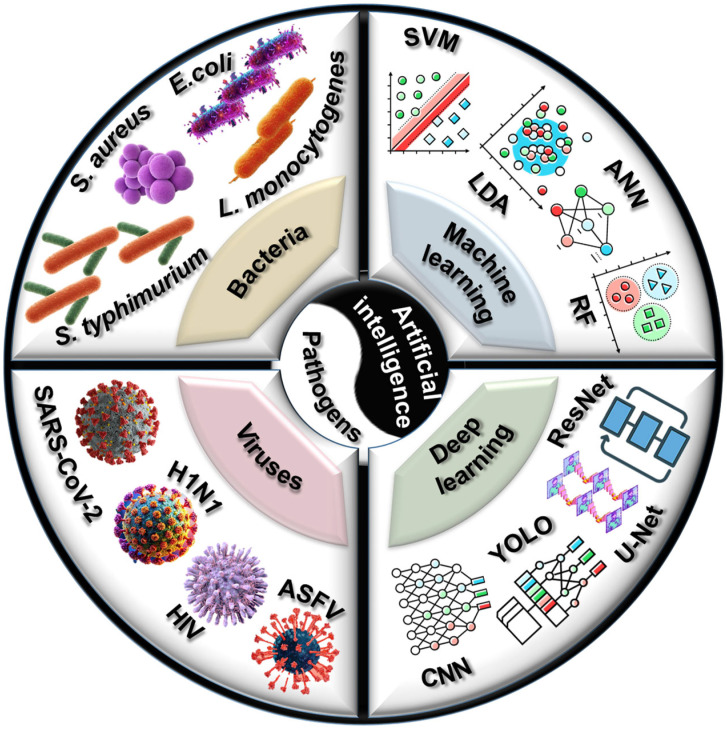
Schematic representation of the integration of artificial intelligence (AI) models with various biosensors for bacterial and viral detection.

Infectious diseases caused by bacterial and viral pathogens remain a major global health challenge in low- and middle-income countries. Rapid, accurate, and accessible diagnostic tools are essential for effective disease control and outbreak prevention. However, conventional diagnostic methods often require sophisticated instrumentation and are prone to operator-dependent variability, which limits their applicability to point-of-care testing (POCT). Pathogens present in food, water, and air pose serious risks to human health, while bacterial diseases such as cholera, pneumonia, tuberculosis, and urinary tract infections (UTIs) continue to cause significant morbidity and mortality worldwide. The World Health Organization (WHO) assessed 1652 pathogens from 28 viral families and a large group of bacteria [1,2,3]. Antimicrobial resistance (AMR) poses a serious threat to global public health and development, contributing to approximately 4.95 million deaths annually and imposing a substantial economic impact, with projected global healthcare costs of US $1 trillion by 2050, according to estimates by the World Bank (Figure 1). Viral pathogens, including coronaviruses, influenza viruses, filoviruses, African swine fever virus (ASFV), and flaviviruses pose additional challenges due to their high transmission and rapid mutation capabilities [4,5]. Coronavirus disease (COVID-19) is caused by severe acute respiratory syndrome coronavirus 2 (SARS-CoV-2), and has resulted in approximately 6.9 million confirmed deaths worldwide since it first emerged in December 2019 [1,6,7]. These factors collectively highlight the urgent need for rapid, reliable, and field-deployable sensing platforms for pathogen detection (Figure 1).

### 1.2. Limitations of Conventional Colorimetric Biosensors

Colorimetric biosensors have attracted considerable attention as a platform due to their simplicity, low cost, and ability to convert biosensor signals into visible color changes. The feature that makes them particularly attractive for rapid and naked-eye POCT applications is their ability to produce a visible color variation that can be used for pathogen detection without the need for sophisticated instruments. Optical techniques such as colorimetry, surface-enhanced Raman spectroscopy (SERS), and fluorescence enable quick and sensitive detection. Compared to other methods, colorimetric assays have been widely used for pathogen detection in medical and environmental applications [8,9,10,11]. Despite their benefits, conventional colorimetric biosensors exhibit lower accuracy in real-world applications. External environmental variables like lighting conditions, background noise, interferences, camera quality, and fabrication inconsistencies can cause signal fluctuations, and visual color interpretation results in user-dependent variability and poor reproducibility. Furthermore, challenges in low-concentration analysis, non-linear response behavior, multiplexing capability, and operation with complex sample images remain unsolved. Although advances in probe design have improved sensitivity, hardware-based optimization alone cannot fully address the data interpretation and robustness limitations.

### 1.3. Artificial Intelligence-Based Solution for Colorimetric Biosensors

#### 1.3.1. Role of Artificial Intelligence in Colorimetric Biosensors

Since Alan Turing’s foundational concepts in the 1980s, artificial intelligence (AI) has emerged as a powerful analytical tool to overcome the limitations of colorimetric biosensors. AI-assisted approaches enable systems to learn, train, and recognize data directly, unlike traditional computer-based analysis, which relies on predefined rules and fixed thresholds without learning from raw data. However, these models do not autonomously adapt and are typically pre-trained. This capability allows AI models to objectively interpret patterns and color information to suppress noise and improve their decision-making accuracy in colorimetric applications. Through image preprocessing, feature extraction, deep learning, and robustness, AI models can make up for things in the environment that change signal levels and color interpretation, such as lighting conditions, background noise, interferences, camera quality, and manufacturing flaws. Recent developments have focused on integrating colorimetric biosensors with AI and the Internet of Things (IoT) for real-time and automated diagnostics. Machine learning (ML) and deep learning (DL) algorithms can significantly enhance real-time colorimetric biosensors, enabling the design of quick and accurate biosensors, even in complex environments affected by lighting variations, background interferences, and heterogeneous samples (Figure 1) [12,13].

#### 1.3.2. ML Algorithms for Colorimetric Biosensors

ML algorithms have been widely employed to capture nonlinear correlations and enhance the reliability of colorimetric biosensor applications. Commonly used ML algorithms include artificial neural networks (ANN), k-nearest neighbors (kNN), linear discriminant analysis (LDA), random forest (RF), and support vector machines (SVM) (Figure 1 and Table 1). ANNs are multilayer perceptron models capable of learning nonlinear relationships between colorimetric images or spectral data for classification and analyte concentration prediction. kNN is a supervised learning method for classification and regression, where unknown samples are assigned to the most common class based on distance metrics such as Euclidean, Manhattan, and Minkowski distances. LDA performs dimensionality reduction and classification by maximizing class separation while minimizing within-class variations. RF employs an ensemble of decision trees for accurate classification and regression, even with complex datasets. SVM constructs optimal separating hyperplanes in multi-dimensional feature spaces for accurate analysis of colorimetric biosensors data’s [2,3,14]. Overall, ML algorithms enhance colorimetric biosensor performance through classification and regression to improve accuracy in pathogen detection and enable the effective handling of large datasets with complex and nonlinear color variation.

#### 1.3.3. DL Algorithms for Colorimetric Biosensors

DL algorithms provide an effective solution for handling large datasets and complex transformations that are challenging for ML algorithms. ML performs well with small datasets (~100–5000), while DL typically requires larger datasets (~500–50,000) to achieve stable training and superior performance. Commonly used DL algorithms include the convolutional neural network (CNN), you only look once (YOLO), U-shaped network (U-Net), residual network (ResNet), and MobileNet algorithms, as shown in Figure 1 and Table 1. CNNs are widely used to learn visual hierarchies from colorimetric images, and YOLO enables real-time bacterial cluster detection with bounding boxes and class probabilities. U-Net is effective in segmenting reactive regions by focusing on important color differences, and ResNet uses residual learning to pick up on deep and complex color differences for accurate classification. MobileNet utilizes depth-wise-separable convolutions to reduce computational complexity while maintaining speed and accuracy, making it ideal for smartphone-based color analysis. Compared with ML, DL algorithms significantly enhance the speed, accuracy, and dataset size of colorimetric biosensors [2,3,14].

#### 1.3.4. Selection Criteria and Workflow for ML and DL Algorithms in Colorimetric Biosensors

**Figure 2:**
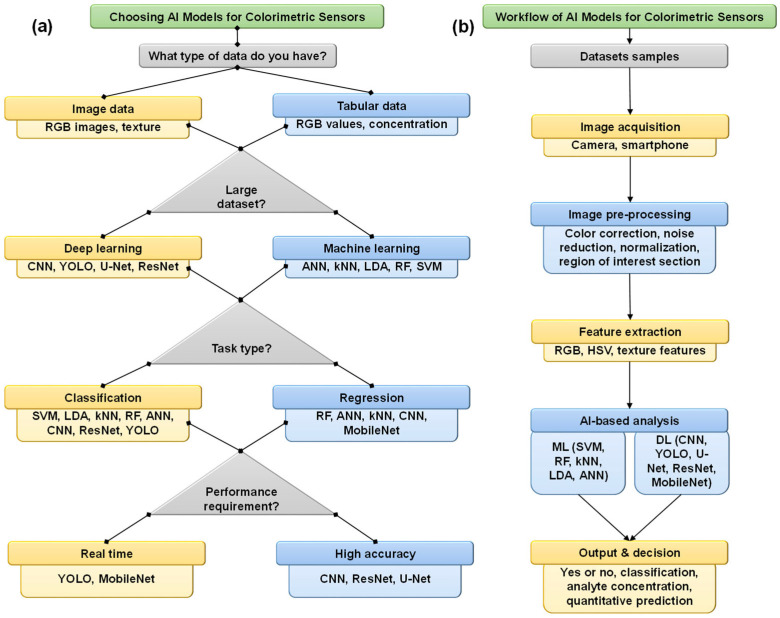
(**a**) Schematic guideline for selecting suitable ML and DL algorithms in an AI-assisted colorimetric biosensor. (**b**) General workflow of an AI-assisted colorimetric biosensor.

The choice between ML and DL algorithms depends on data requirements, application conditions, and practical considerations. For data requirements and application condition perspectives, ML models operate with moderately sized datasets and are suited for well-structured numerical and categorical features with clear definitions. RF and SVM perform well with structured, high-dimensional data but tend to underperform with unstructured data. In contrast, DL models require large datasets for image processing tasks, excelling in recognizing complex patterns that require detailed and pixel-level annotations. CNN and YOLO are tailored for image applications that involve real-time detection and segmentation, though they demand significant computational resources as data size increases. For practical considerations, ML models are hard to run and need to be carefully tuned, especially SVM and ANN models. These models are for simpler, smaller-scale tasks with defined datasets. In contrast, DL models achieve high accuracy with complex tasks but require heavy computational power and graphics processing units for efficiency. MobileNet represents a lightweight DL alternative for resource-limited devices, balancing performance and computer efficiency. Figure 2a and Table 1 show general rules for picking the right ML and DL algorithms for AI-assisted colorimetric biosensors based on the type of data, the size of the data, the needs of the task, and where the sensors will be used. Figure 2b shows the general workflow of an AI-assisted colorimetric biosensor, in which the sensor response is captured via a camera or smartphone. The images use preprocessing, which involves region of interest (ROI), color correction, noise reduction, and normalization. Key features such as color information, texture, red–green–blue (RGB), and hue–saturation–value (HSV) are analyzed using ML and DL models to enable accurate classification and prediction of analyte concentration.

#### 1.3.5. Explainable AI for Transparent Colorimetric Biosensors

Table 1 indicates that ML and DL algorithms are predominantly implemented as supervised models, where classical ML models typically use handcrafted color features and DL models mainly operate on raw images and are often task-specific. Despite their strong predictive performance, many ML and DL models used in colorimetric biosensors are often criticized for their “black box” nature, where the decision-making process lacks transparency. This limitation can hinder clinical trust and real-world applications. To address this concern, explainable AI (XAI) techniques identify critical color features, image regions, and sensor responses that influence the output. Feature importance analysis and XAI tools such as SHapley Additive exPlanation (SHAP) and Local Interpretable Model-agnostic Explanations (LIME) can explain ML decisions using RGB and HSV features. Gradient-weighted Class Activation Mapping (Grad-CAM) can highlight critical regions in DL-based colorimetric images. The integration of XAI improves the reliability, accuracy, and interpretability of the AI-assisted colorimetric biosensor.

### 1.4. Scope of This Review

Although a few review articles have addressed conventional colorimetric and AI-based analytical methods, a comparative, challenge-driven review that critically integrates existing limitations with AI-based solutions remains limited. In this review, we present critical problems and limitations in conventional colorimetric biosensors and the role of ML and DL algorithms in addressing these issues. Our scope focuses on AI-assisted colorimetric biosensors for bacterial and viral diagnostics, such as recent viral studies that are often overlooked in existing reviews. Recent advances over the past five years are summarized, and future perspectives are proposed for AI-assisted platforms for accurate and on-site pathogen diagnostics. Moreover, we provide an end-to-end workflow and include model-wise subdiscussions of major ML and DL approaches supported by relevant studies. The following section explores the pros and cons of colorimetric biosensors based on ML and DL.

## 2. AI-Assisted Colorimetric Detection of Bacteria

Colorimetric biosensors are cost-effective and easily accessible analytical tools for bacterial detection, providing a naked-eye visual readout without the need for sophisticated instrumentation. Integration of AI with colorimetric biosensors enables automated, accurate, quantitative, and rapid interpretation. However, AI models do not autonomously adapt and are typically pre-trained. This section provides an overview of ML and DL algorithms that enhance the performance of colorimetric biosensors for bacterial detection.

### 2.1. ML-Based Colorimetric Detection of Bacteria

Image-based collection, processing, and analysis of colorimetric biosensor data involve extracting color information such as RGB, HSV, hue–saturation–lightness (HSL), and cyan–magenta–yellow–key (CMYK) values. The relationship between color changes and bacterial concentrations may be influenced by environmental factors, including background noise, environmental lighting, and the reagents used. ML algorithms have been employed to capture these nonlinear correlations and enhance the reliability of bacterial detection. ML-based colorimetric biosensors enable the automated processing of data with high accuracy for determining bacterial concentrations. In recent years, several ML algorithms such as ANN, kNN, LDA, RF, and SVM have been widely used for the colorimetric detection of bacteria.

#### 2.1.1. ANN for Bacterial Detection

**Figure 3:**
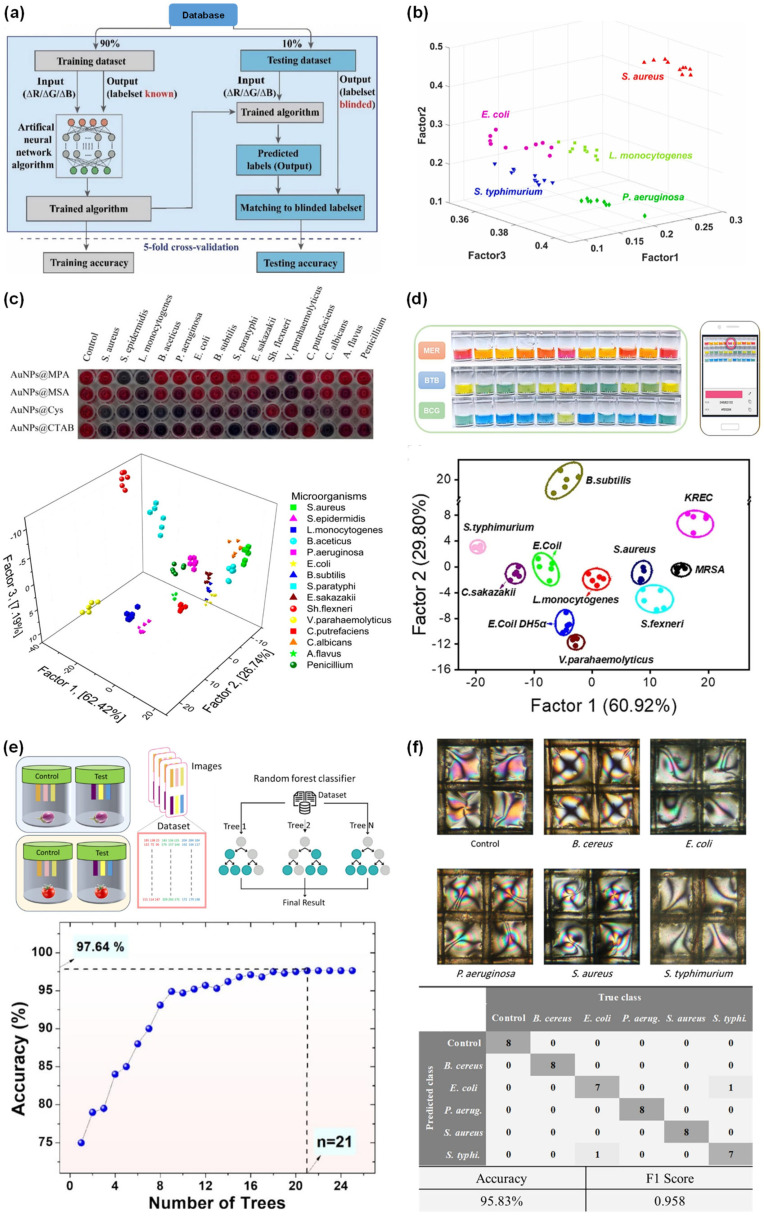
(**a**) Schematic illustration of ANN-based analysis; reprinted with permission from [16], copyright 2024 Elsevier. (**b**) Multibacterial sensing analysis illustrated using kNN-based classification; reprinted with permission from [19], copyright 2025 Elsevier. (**c**) Colorimetric responses and LDA-based analysis of 15 bacteria using gold nanoparticles@ligand sensor array; reprinted with permission from [20], copyright 2017 American Chemical Society (ACS). (**d**) Colorimetric response and LDA-based analysis of 11 bacteria using methyl red, bromothymol blue, and bromocresol green sensor arrays; reprinted with permission from [24], copyright 2024 Elsevier. (**e**) Schematic workflow and RF-based model showing spoilage level detection in vegetables; reprinted with permission from [28], copyright 2025 Elsevier. (**f**) Photographs of bacterial strains and their classification using SVM analysis; reprinted with permission from [33], copyright 2024 ACS.

ANN is a multilayer perceptron model that learns nonlinear relationships between colorimetric images and spectral data for detection tasks. ANN processes colorimetric information such as color data, bacterial species detection, and concentration estimation. Recent advancements have led to the development of novel ANN-based colorimetric biosensing platforms for detecting food pathogens. This approach was demonstrated by Jia et al. (2024) [15], who used machine learning to make a paper chromogenic array (PCA) system that could continuously perform non-contact monitoring for *Escherichia coli* (*E. coli*) *O157:H7*, *Salmonella*, and *Listeria monocytogenes* (*L. monocytogenes*) in chicken samples. The detection method relied on exposure to volatile organic compounds (VOCs) released by contaminated chicken samples. These VOCs interacted with a PCA containing colorimetric dyes, causing visible color shifts. The color changes were converted into digital color data for optimized ANN model analyses and the accurate identification of the respective bacteria. The ANN model detected bacteria at a limit of detection (LOD) of 10 colony-forming units (CFUs)·g^−1^ with 90% accuracy, enabling real-time food safety monitoring [15]. In the same way, Jia et al. (2024) [16] developed an ANN–PCA method for finding *E. coli O157:H7* and *Salmonella enteritidis* (*S. enteritidis*) in shredded cheddar cheese in real time. The ANN used a deep feedforward network with several layers and 1024 nodes per hidden layer to look at color changes related to the dye-infused PCA assay induced by VOCs. This system showed greater noise tolerance and classification robustness than conventional ML algorithms. Specifically, the system achieved an LOD of 10 CFUs·g^−1^ with 92% accuracy without enrichment, enabling real-time food safety monitoring (Figure 3a) [16]. Overall, these studies demonstrate the effectiveness of ANN in extracting nonlinear correlations from optical colorimetric datasets. Because ANN can handle complex and overlapping signals even when there is interference, it is well-suited for next-generation biosensors used in POCT and food safety (Table 2). The PCN-ANN systems developed by Jia et al. (2024) [15,16] enable non-contact and continuous VOC-based monitoring of pathogens in real food samples. However, they are sensitive to local volatile environmental conditions and therefore require careful calibration and transfer learning.

**Table 2:** Overview of ML and DL algorithms for colorimetric biosensor-based detection of bacteria.

Sl. No.	Category/AI Algorithm	Target Bacteria	Data Size	Accuracy	LOD/Linear Range	Reaction Time	Real Samples	Ref.
1	ML/ANN	*E. coli O157:H7*, *Salmonella*, and *L. monocytogenes*	500	90%	10 CFUs·g^−1^/N.A.	N.A.	Chicken	[15]
2	ML/ANN	*E. coli O157:H7* and *S. enteritidis*	200	92%	10 CFUs·g^−1^/N.A.	24 h	Shredded cheddar cheese	[16]
3	ML/kNN	*E. coli*, *S. typhimurium*, *S. aureus*, *P. aeruginosa*, *Shigella*, and *L. monocytogenes*	200	100%	92–121 CFUs·mL^−1^/N.A.	60 min	Tap water	[17]
4	ML/kNN	*S. typhimurium*, *E. coli*, *S. aureus*, *P. aeruginosa*, and *L. monocytogenes*	75	98%	10^3^ CFUs·mL^−1^/10^3^–10^7^ CFUs·mL^−1^	5 min	Tap water and milk	[18]
5	ML/kNN	*E. coli*, *P. aeruginosa*, *S. aureus*, *S. typhimurium*, and *L. monocytogenes*	50	80%	10^3^ CFUs·mL^−1^/10^3^–10^7^ CFUs·mL^−1^	30 min	Tap water	[19]
6	ML/LDA	*S. aureus*, *S. epidermidis*, *L. monocytogenes*, *B. aceticus*, *P. aeruginosa*, *E. coli*, *B. subtilis*, *S. paratyphi*, *E. sakazakii*, *S. flexneri*, *V. parahemolyticus*, *C. putrefaciens*, *C. albicans*, *A. flavus*, and *Penicillium*	60	100%	N.A./N.A.	5 s	N.A.	[20]
7	ML/LDA	*B. subtilis*, *E. coli*, *S. typhimurium*, kanamycin-resistant *E. coli* (KREC), methicillin-resistant *S. aureus* (MRSA), *S. aureus*, *V. parahaemolyticus*, *S. flexneri*, and *C. sakazakii*	180	100%	N.A./10^3^–10^6^ CFUs·mL^−1^	30 min	Tap water	[21]
8	ML/LDA	*S. aureus*, *Salmonella*, *V. vulnificus*, *V. harvey*, *L. monocytogenes*, and *V. parahaemolyticus*	72	95%	N.A./10^5^–10^8^ CFUs·mL^−1^	10 min	Sea water, lake water, tap water, and coconut water	[22]
9	ML/LDA	*S. aureus*, *E. coli*, *S. typhimurium*, *S. senftenberg*, *L. monocytogenes*, *S. epidermidis*, and *B. subtilis*	80	100%	N.A./10^3^–10^7^ CFUs·mL^−1^	50 min	Serum and urine	[23]
10	ML/LDA	MRSA, *L. monocytogenes*, *E. coil*, *S. flexneri*, KREC, *S. typhimurium*, *B. subtilis*, *S. aureus*, *C. sakazakii*, and *V. parahaemolyticus*	50	100%	N.A./10^3^–10^7^ CFUs·mL^−1^	6 h	Tap water	[24]
11	ML/LDA	*S. aureus*, *E. coli*, and *E. faecalis*	50	100%	10^5^ CFUs·mL^−1^/N.A.	20 min	Urine	[25]
12	ML/LDA	*E. coli*, *S. typhimurium*, *E. sakazakii*, *P. aeruginosa*, *S. aureus*, and *L. monocytogenes*	150	93.3%	N.A./N.A.	15 min	Milk	[26]
13	ML/RF	*E. coli* and *S. epidermidis*	320	97%	10 CFUs·mL^−1^/10^2^–10^7^ CFUs·mL^−1^	15 min	Tap water, sea water, and artificial saliva	[27]
14	ML/RF	*E. coli*, *P. aeruginosa*, *S. aureus*, and *P. syringae*	300	97.64%	N.A./N.A.	30 min	Vegetables	[28]
15	ML/SVM	*E. coli* and *S. typhimurium*	N.A.	N.A.	10^3^ CFUs·mL^−1^/10^2^–10^8^ CFUs·mL^−1^	30 min	Pear juice	[29]
16	ML/SVM	*E. coli*, *S. aureus*, *S. typhimurium*, *E. faecium*, and *P. aeruginosa*	N.A.	93.3%	10^5^ CFUs·mL^−1^/N.A.	10 min	Pond water	[30]
17	ML/SVM	*E. coli*, *K. aerogenes*, *P. aeruginosa*, *P. vulgaris*, *E. faecalis*, *E. faecium*, *S. aureus*, *S. epidermidis*, *M. albican*, and *C. glabrata*	60	97%	10^4^ CFUs·mL^−1^/N.A.	60 min	Urine	[31]
18	ML/SVM	*C. sakazakii*, *S. enteritidis*, *L. monocytogenes*, *V. parahaemolyticus*, *S. aureus*, *S. dysenteriae*, *C. jejuni*, and *E. coli*	300	93.75%	10^2^ CFUs·mL^−1^/10^2^–10^7^ CFUs·mL^−1^	60 min	Milk	[32]
19	ML/SVM	*B. cereus*, *E. coli*, *P. aeruginosa*, *S. aureus*, and *S. typhimurium*	N.A.	86.58–97.92%	10 CFUs·mL^−1^/10–10^6^ CFUs·mL^−1^	20 min	Drinking water, milk, and apple juice	[33]
20	DL/CNN-SVM	*E. coli*, *P. aeruginosa*, and *S. aureus*	1000	96.2%	10 CFUs·mL^−1^/10–10^3^ CFUs·mL^−1^	24 h	Human blood	[34]
21	DL/YOLO	*E. coli*, *P. aeruginosa*, *S. aureus*, and Group A *Streptococcus*	1419	92%	10 CFUs·mL^−1^/10–10^5^ CFUs·mL^−1^	60 min	Blueberry	[35]
22	DL/YOLO	*E. coli*, *S. pneumoniae*, and *H. influenzae*	500	97.8%	6.25 fmol/0–500 fmol	15 min	N.A.	[36]

Abbreviations: CFU, colony-forming unit; LOD, limit of detection; N.A., not available; No., number; Ref., reference.

#### 2.1.2. kNN for Bacterial Detection

The kNN algorithm is a simple and powerful supervised learning approach that is widely used for both classification and regression tasks. In the field of biosensors, kNN algorithms are frequently used to classify bacterial species by analyzing the optical colorimetric outputs from sensor assays. Recent literature supports the growing adoption of this method in optical techniques such as fluorescence and colorimetry. Zhang et al. (2025) [17] developed perovskite quantum dot (PQD)-based fluorescent sensors that work with a kNN algorithm to find different types of bacteria, including *E. coli*, *Salmonella typhimurium* (*S. typhimurium*), *Staphylococcus aureus* (*S. aureus*), *Pseudomonas aeruginosa* (*P. aeruginosa*), *Shigella*, and *L. monocytogenes*. Green-, cyan-, and blue-emitting PQDs synthesized via halide ion exchange exhibited strong electrostatic interactions with bacterial membranes and showed enhanced fluorescence quenching. Smartphone-captured RGB data were analyzed using principal component analysis and subsequently classified by kNN, achieving 100% accurate recognition of *E. coli*, *S. typhimurium*, *S. aureus*, *P. aeruginosa*, *Shigella*, and *L. monocytogenes* [17]. Similarly, Zhang et al. (2025) [18] developed an antibiotic-functionalized nitrogen- and sulfur-doped graphene quantum dot (NS-GQD) array for the broad-spectrum detection of multiple bacterial species such as *S. typhimurium*, *E. coli*, *S. aureus*, *P. aeruginosa*, and *L. monocytogenes*. Antibiotic functionalization of NS-GQD using spectinomycin, kanamycin, and polymyxin B showed fluorescence-quenching behavior dependent on the bacterial surface charge and structure. The kNN model achieved 98% accuracy, an LOD of 10^3^ CFUs·mL^−1^, and a linear range of 10^3^–10^7^ CFUs·mL^−1^ [18]. In 2025, Zhu et al. [19] reported a paper-based fluorescence sensor array that used silver-, copper-, and zinc-doped carbon quantum dots to simultaneously detect and kill multiple bacterial species. These bacteria included *E. coli*, *P. aeruginosa*, *S. aureus*, *S. typhimurium*, and *L. monocytogenes*. The kNN algorithms were applied to quantify fluorescence color quenching when bacteria interact with these through aggregation-induced quenching and with smartphones that used calculated color data. A linear range of 10^3^–10^7^ CFUs·mL^−1^ within 30 min was observed (Figure 3b) [19]. Overall, these results demonstrate that kNN is a robust and data-efficient algorithm with low computational complexity for bacterial identification in colorimetric biosensor platforms (Table 2).

Overall, kNN is easy to interpret but struggles with larger datasets, high-dimensional feature spaces, and noisy data, which reduce reliability in real-sample applications. Across these studies, kNN’s performance depends strongly on feature separability and signal stability. The PCA-assisted PQA system reported by Zhang et al. (2025) [17] achieved the highest accuracy (100%) and supported larger datasets because the color pattern was well defined. On the other hand, the antibiotic-functionalized NS-GQD array developed by Zhang et al. (2025) [18] was more reliable in real samples but exhibited lower accuracy. In contrast, the multifunctional sensing platform proposed by Zhu et al. (2025) [19] exhibited increased signal variability and a limited dataset size, resulting in low classification reliability and overfitting.

#### 2.1.3. LDA for Bacterial Detection

The LDA algorithm is a supervised method for transforming high-dimensional data into low-dimensional data to maximize the separation between classes and minimize variance within classes. LDA performs effectively with multispectral absorbance and color datasets derived from nanomaterials (NMs) and enzyme-mimicking sensor arrays, a concept demonstrated in several biosensor studies. For example, Li et al. (2017) [20] reported a colorimetric sensor array based on gold nanoparticles (AuNPs) coated with mercaptopropionic acid, mercaptosuccinic acid, cysteamine, and cetyltrimethylammonium bromide. The integrated LDA colorimetric sensor system identified 12 bacteria and 3 fungi within 5 s with 100% accuracy through visible color shifts due to electrostatic and hydrophobic interactions (Figure 3c) [20]. Nanozymes are synthetic NMs with catalytic activities similar to those of bioenzymes. Nanozymes are widely used in biosensing, cancer treatment, and environmental remediation. Similarly, Zhao et al. (2022) [21] introduced 3,3′,5,5′-tetramethylbenzidine (TMB)-functionalized palladium/platinum bimetallic NP nanozymes for simultaneous bacterial identification and photothermal inactivation. Based on the changes in four absorbance peaks of oxTAMP, LDA can be used to accurately detect and monitor the following pathogens such as *Bacillus subtilis* (*B. subtilis)*, *E. coli*, *S. typhimurium*, kanamycin-resistant *E. coli* (KREC), methicillin-resistant *S. aureus* (MRSA), *S. aureus*, *Vibrio parahaemolyticus* (*V. parahaemolyticus*), *Shigella flexneri* (*S. flexneri*), and *Cronobacter sakazakii* (*C. sakazakii*). OxTMB functions as both a recognition signal and a photothermal, acting as a dual-function LDA-integrated nanozyme for clinical applications [21]. Similarly, nanozyme results have confirmed the utility of Fe–N–C single-atom assay-based colorimetric biosensors. A peroxidase-like nanozyme-catalyzed substrate, such as TMB, 2,2′-azino-bis (3-ethylbenzothiazoline-6-sulfonic acid), and o-phenylenediamine, can be used to produce different colors, whose variations across different sizes of foodborne pathogens were analyzed by LDA. LDA exhibited clear cluster boundaries for species such as *S. aureus*, *Salmonella*, *Vibrio vulnificus* (*V. vulnificus*), and *L. monocytogenes*, achieving 95% accuracy with a linear detection range of 10^5^–10^8^ CFUs·mL^−1^ within 10 min. The results showed that combining an LDA-assisted colorimetric assay with nanozyme catalysis enables effective pathogen detection in tap, lake, and seawater samples [22]. In another innovative study, Lin et al. (2025) [23] introduced a metabolism-derived “read-to-answer” AuNPs sensor assay, where bacterial catalase activity mediated hydrogen peroxide (H_2_O_2_)-induced NP changes, producing color changes in images and spectral data. The LDA achieved 100% accuracy across seven bacterial species, such as *S. aureus*, *E. coli*, *S. typhimurium*, *Salmonella senftenberg* (*S. senftenberg*), *L. monocytogenes*, *Staphylococcus epidermidis* (*S. epidermidis*), and *B. subtilis*. The LDA-based colorimetric assay enabled simultaneous bacterial identification and antimicrobial-susceptibility testing (AST) directly from serum and urine samples [23]. It was reported by Zhou et al. (2024) [24] that they used a glucose metabolism-driven colorimetric POCT sensor assay with pictogram-sensitive dyes such as methyl red, bromothymol blue, and bromocresol green to identify bacteria and measure AST. Different bacterial metabolic pathways generate different acidic byproducts during glucose metabolism. These byproducts enhance pH-dependent color changes in the different dye assays. The LDA-derived color values were used to classify bacterial species and quantify antibiotic-dependent inhibition. LDA enabled the identification of multiple bacterial species, including *MRSA*, *L. monocytogenes*, *E. coli*, *S. flexneri*, KREC, *S. typhimurium*, *B. subtilis*, *S. aureus*, *C. sakazakii*, and *V. parahaemolyticus*, with a linear range of 10^3^–10^7^ CFUs·mL^−1^ and 100% accuracy. Furthermore, these sensors successfully identified drug-resistant strains (MRSA and KREC) in both tap water and milk samples (Figure 3d) [24]. To map antibiotic-response spectra and quickly identify different bacteria, Wang et al. (2024) [25] created a metabolic labeling-assisted “chemical nose” array that combined amino acid tagging with dibenzocyclooctyne-functionalized up-conversion nanoparticles (DBCO-UCNPs). Bacterial samples were metabolically labeled with 3-azido-d-alanine in the presence or absence of antibiotics, including tetracycline, oxacillin, polymyxin B, vancomycin, levofloxacin, and cefepime. DBCO-UCNP conjugation via click chemistry reflected bacterial metabolic activity. The color intensity was analyzed using LDA to identify bacterial species. The LDA achieved 100% accuracy with an LOD of 10^5^ CFUs·mL^−1^ in an artificial urine sample. This study broadened LDA applications from morphological pattern recognition to phenotype-metabolic sensing, facilitating simultaneous detection and AST [25]. Wang et al. (2023) [26] applied an LDA framework to a single-probe dual-mode colorimetric–photothermal bacterial sensor array using boronic acid-functionalized Au-iron oxide nanoparticles, (Au-Fe_3_O_4_) NPs, for simultaneous thermal and colorimetric detection of multiplex pathogens. Au-Fe_3_O_4_ NPs can covalently bind cis-diol groups on bacterial cell walls, thereby controlling sodium chloride-induced NPs aggregation based on the different bacterial surface-induced NPs stabilities, colors, and photothermal properties. Color and temperature shifts were jointly analyzed via LDA, enabling the identification of multiple bacterial species. The dual-mode LDA array collectively processed hybrid optical and thermal datasets to enhance accuracy [26].

Overall, the LDA-integrated biosensing platform effectively transforms complex multidimensional optical responses into accurate bacterial identification for next-generation intelligent POCT and AMR diagnostics (Table 2). However, LDA struggles with high-dimensional and non-linear data, which limits its use in complex applications. Across these, Li et al. (2017) [20] achieved the fastest and most accurate performance (5 s, 100%) for multi-pathogen sensing but remained sensitive to nanoparticle variability. Nanozyme-based Fe–N–C assays reported by Zhao et al. (2022) [21] enhanced signal richness via multichannel color output through accuracy constrained by dataset size and environmental variability. The metabolism-guided and phenotype-labeling dual-mode methods reported by Lin et al. (2025) [23], Zhou et al. (2024) [24], Wang et al. (2024) [25], and Wang et al. (2023) [26] extended LDA beyond simple identification to enable AST with longer assay times and complex protocol control. However, the reports by Wang et al. (2023) [26], Zhou et al. (2024) [24], Wang et al. (2024) [25], and Li et al. (2017) [20] were conducted with small datasets and lacked parameters like LOD and concentration, which limited their robustness. Overall, LDA functions primarily as a rapid and interpretable classification method rather than a nonlinear modeling approach, and the platform reported by Li et al. (2017) [20] is best suited for ultrafast diagnostics and future POCT applications.

#### 2.1.4. RF for Bacterial Detection

The RF algorithm is a strong ML method that employs decision trees to accurately classify and regress nonlinear, multidimensional datasets. RF is particularly useful for tasks such as gene expression analysis and color-image identification. In a notable application, Pennacchio et al. (2024) [27] reported AuNPs functionalized with a hydrophobin–antimicrobial peptide chimera for rapid and portable colorimetric bacteria detection. The Vmh2–GKY20 chimera was anchored onto AuNPs, where bacterial binding modulated NPs aggregation under phosphate-buffered saline-induced charge neutralization, resulting in visible color shifts related to the bacterial concentration. RF, extreme gradient boosting (XGBoost), and multilayer perceptron were employed to classify color images in the assay wells and determine their bacterial concentrations. The integrated RF achieved 97% classification accuracy and an LOD of 10 CFUs·mL^−1^, enabling smartphone-based quantification without a dedicated reader [27]. Acharyya et al. (2025) [28] introduced a paper-based colorimetric platform using pH-indicator dyes for visual, nondestructive detection of bacterial contamination in vegetables. The VOCs emitted by metabolically active bacteria interacted with pH-sensitive dyes (chlorophenol red, methyl red, and bromocresol purple) to enhance proton exchange and color changes. The RF model achieved 97.64% accuracy in classifying bacteria and monitoring spoilage across multiple vegetables, detecting contamination within 30 min (Figure 3e) (Table 2) [28].

Both reports demonstrated that integrating RF algorithms with colorimetric biosensors enhanced the detection accuracy at a low cost and rapidly identified bacteria for real-world food safety and POCT applications. Pennacchio et al. (2024) [27] achieved sensitive POCT detection in AuNP aggregation, reaching a low LOD with 97% accuracy without a dedicated instrument. In contrast, Acharyya et al. (2025) [28] show VOC-driven spoilage detection in vegetables with the same accuracy, but their methods are constrained by a higher response time and data unavailability. Overall, Pennacchio et al. (2024) [27] is best suited for high-sensitivity detection for liquid-phase samples in future works.

#### 2.1.5. SVM for Bacterial Detection

The SVM algorithm finds the best separating hyperplanes in multiple-dimensional feature spaces. This enables biosensors to accurately classify optical or flow-based patterns. Somvanshi et al. (2022) [29] demonstrated a microfluidic paper-based aptasensor for detecting *E. coli* and *S. typhimurium* using multiple colorimetric methods. Aptamer-modified AuNP-polystyrene particles (PSPs) aggregated upon salt exposure, which enhanced target-specific color changes that were measured using image analysis. The SVM algorithm was applied to extract CIELAB (Lab) color features to identify and quantify *E. coli* and *S. typhimurium*. This SVM system demonstrated sensitive detection with an LOD of 10^3^ CFUs·mL^−1^, a linear range of 10^2^–10^8^ CFUs·mL^−1^, and accurate multiplex identification in buffer and pear juice samples [29]. Kim et al. (2021) [30] developed a peptide-mediated paper-based microfluidic platform for the identification of multiple bacteria, such as *E. coli*, *S. aureus*, *S. typhimurium*, *Enterococcus faecium* (*E. faecium*), and *P. aeruginosa*. Peptide–bacteria associated with PSP particle aggregation modulated the flow velocity, which was recorded by a smartphone. SVM analysis identified multidimensional flow-velocity fingerprints generated by different peptides and bacteria combinations. This model achieved 93.3% accuracy across multiple species with a 10 min total assay time [30]. An iron single-atom nanozyme-based colorimetric sensor array was designed to identify microorganisms in samples from patients with UTIs. Single-atom nanozymes were functionalized to interact with multiple pathogens and generate specific colorimetric patterns revealing inhibition during TMB oxidation. An SVM algorithm was used to analyze U-MAP-reduced colorimetric features to identify both bacterial and fungal samples. This AI-based model achieved up to 97% accuracy using 60 clinical UTI samples and detected 10 microbial species within 60 min [31]. Li et al. (2024) [32] reported ML-assisted aggregation-induced emissive nanosilicon-based sensor arrays for POCT identification of multiple food-borne pathogens such as *C. sakazakii*, *S. enteritidis*, *L. monocytogenes*, *V. parahaemolyticus*, *S. aureus*, *Shigella dysenteriae* (*S. dysenteriae*), *Campylobacter jejuni* (*C. jejuni*), and *E. coli*. Four types of tetraphenylethylene-doped silicon NPs with distinct surface chemistries and hydrophobic properties interacted with individual bacterial surfaces to enhance the fluorescence color fingerprints. Multi-ML algorithms such as SVM, LDA, RF, ANN, and XGBoost were used to analyze variations in fluorescence color for multidimensional pathogen identification. The SVM-based sensor array detected 8 pathogens within 60 min with 93.75–100% accuracy [32]. Mousavizadegan et al. (2024) [33] reported a single-probe liquid crystal optical sensor array enhanced with cysteine-functionalized silver NPs (AgNPs) for the simultaneous detection of food- and water-borne bacteria such as *Bacillus cereus* (*B. cereus*), *E. coli*, *P. aeruginosa*, *S. aureus*, and *S. typhimurium*. Interactions between bacteria and AgNPs modulated the liquid crystal orientation, producing specific optical properties under light. The SVM classifier was trained on extracted greyscale, RGB, HSV, and color features to recognize multiple bacteria. The SVM delivered 98.89% accuracy in identifying five bacteria species with an LOD of 10 CFUs·mL^−1^ and real sample prediction accuracies of 95.83% (drinking water), 97.92% (apple juice), and 86.58% (milk) (Figure 3f) (Table 2) [33].

Overall, these reports indicate that SVM is a robust classifier that can accurately interpret complex optical, flow-based, and fluorescence-based biosensors in POCT applications. Microfluidic paper systems enable rapid screening, as reported by Kim et al. (2021) [30], achieving the fastest response within 10 min, and nanozyme-based clinical platforms show high accuracy for complex samples. Mousavizadegan et al. (2024) [33] achieved the highest analytical sensitivity and accuracy performance in protein-rich milk. Overall, the method proposed by Kim et al. (2021) [30] is best suited for rapid microfluidic sensing applications.

### 2.2. DL-Based Colorimetric Detection of Bacteria

ML algorithms have been widely employed to improve the analysis of colorimetric data by enabling the automated extraction and interpretation of color features. However, as datasets grow larger and more complex, variations in color and irregular bacterial growth pose challenges for maintaining accuracy and robustness in ML algorithms. In contrast, DL algorithms can automatically learn complex patterns and nonlinear features from colored images more effectively than ML algorithms, even though they do not autonomously adapt and are typically pre-trained. DL-based colorimetric biosensors enable highly accurate, rapid, and remarkable determinations of bacterial concentrations. Among the various DL algorithms, CNN and YOLO have demonstrated notable performance in colorimetric bacterial sensing applications.

#### 2.2.1. CNN for Bacterial Detection

**Figure 4:**
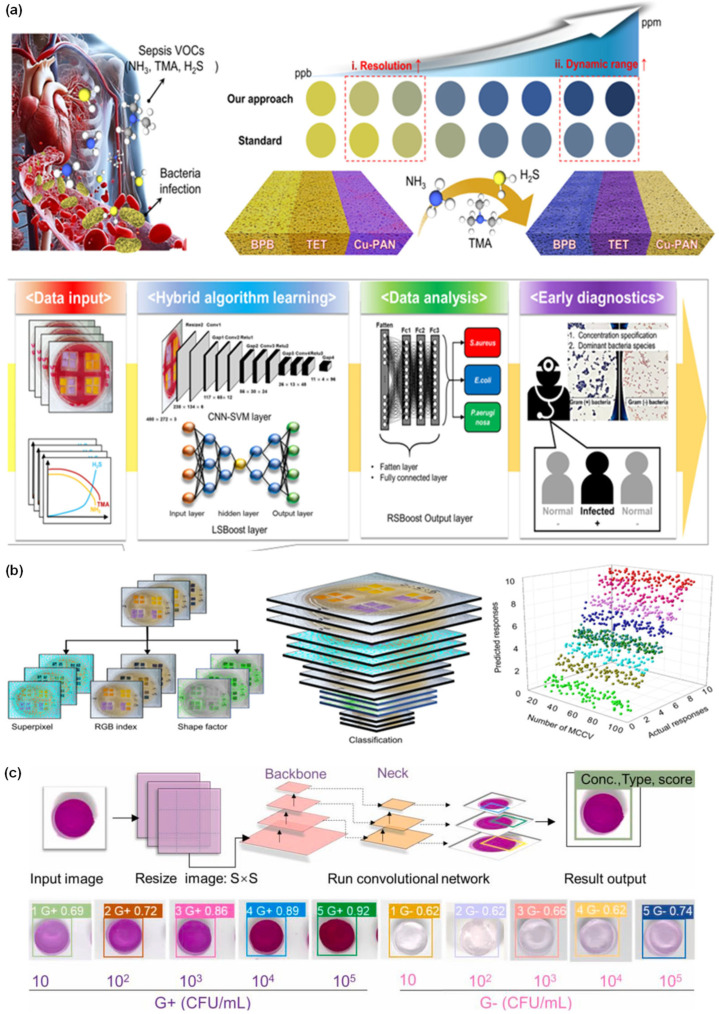
(**a**) Schematic diagram depicting a hybrid CNN–SVM–least-squares boosting (LSBoost) algorithm for identifying sepsis-associated bacteria; reprinted with permission from [34], copyright 2025 Springer Nature. (**b**) Hybrid CNN–SVM–LSBoost framework illustrated by a 3-dimensional scatter plot for *S. aureus*, *E. coli*, and *P. aeruginosa* showing classification results and accuracy; reprinted with permission from [34], copyright 2025 Springer Nature. (**c**) Flowchart illustrating YOLO-based detection of Gram-positive and Gram-negative bacteria; reprinted with permission from [35], copyright 2024 Elsevier.

CNN algorithms automatically learn hierarchical visual features and are therefore particularly powerful for biosensors and clinical diagnosis. Shin et al. (2025) [34] demonstrated this capability by combining a colorimetric gas sensor array with an AI-based hybrid model to enable rapid image analysis for detecting sepsis-causing bacteria based on VOC emission features. Time-resolved sensor images were analyzed to identify distinct morphological color evolution patterns in each bacterial sample. The images used as input for the CNN layer included spatial features such as length, color, and shape over a 24 h period. The CNN–SVM hybrid model converted extracted features into an activation map and then performed multiclass classification to accurately distinguish *E. coli*, *P. aeruginosa*, and *S. aureus*. The hybrid CNN–SVM model achieved 96.2% diagnostic accuracy for human blood samples (Figure 4a,b) [34]. The results demonstrated that deep feature extraction combined with a classical ML classifier enhanced accuracy, accelerated analysis, and improved reliability for bacterial detection in clinical applications (Table 2). However, the CNN–SVM model layer’s dependence on prolonged (24 h) VOC image acquisition limits its suitability for rapid sepsis diagnostics.

#### 2.2.2. YOLO for Bacterial Detection

The YOLO algorithm is a real-time detection network that simultaneously simplifies and classifies regions of interest during a single forward pass. In biosensing applications, YOLO enables the detection of bacterial colonies and assay reaction zones using smartphone images. Cui et al. (2024) [35] developed a smartphone-based colorimetric biosensor to evaluate bacterial hyaluronidase activity using a β-d-galactopyranoside (β-gal) hydrogel and a chlorophenol red-β-d-galactopyranoside (CPRG)-loaded hyaluronic acid (HA) bioreactor. Bacterial hyaluronidase degraded the HA hydrogel, releasing CPRG, which subsequently reacted with the β-gal hydrogel to generate concentration-dependent color changes that were captured by a smartphone. A custom YOLOv5 model was trained on 1419 images to automatically detect bacterial types and concentrations based on reaction zones and color features. YOLOv5 achieved 92% accuracy and enabled an LOD of 10 CFUs·mL^−1^ within a total assay time of 60 min with blueberry samples and effectively identified both Gram-positive and Gram-negative bacteria (Figure 4c) [35]. Papadopoulos et al. (2025) [36] used a smartphone application powered by machine vision to analyze multicolor lateral flow assay (LFA) strips for the simultaneous detection of *E. coli*, *Streptococcus pneumoniae* (*S. pneumoniae*), and *Haemophilus influenzae* (*H. influenzae*). Different bacterial species produced distinct color signals on bead probes in a multianalyte LFA strip, which were captured using a smartphone. The YOLOv8 model enabled robust strip localization under variable lighting and backgrounds and served the extracted signal regions into a CNN-based model. The YOLOv8-CNN hybrid model accurately detected multiple pathogens and reliably recognized strips at various angles and automatically interpreted four infectious disease biomarkers (Table 2) [36].

Overall, these results show that YOLO-based models have significantly improved biosensor automation by enabling smartphone imaging for quick, accurate, and angle-independent bacterial detection. However, YOLO models can produce false positives in complex environments and require large labeled datasets for effective training. Cui et al. (2024) [35] demonstrated a YOLOv5-assisted enzymatic colorimetric assay that achieved sensitive and quantitative detection. Papadopoulos et al. (2025) [36] employed a YOLOv8-CNN hybrid model to obtain reliable multiplex LFA readouts, making it well-suited for field applications. Common challenges include imaging variability, low dataset size, and model transferability. Overall, the approach reported by Cui et al. (2024) [35] is best for quantitative POCT, whereas the method proposed by Papadopoulos et al. (2025) [36] is most suitable for robust onsite diagnostics.

## 3. AI-Assisted Colorimetric Detection of Viruses

Colorimetric biosensors have emerged as a simple and popular analytical method for detecting viruses, offering rapid results without the need for sophisticated instrumentation. However, attempts to manually detect subtle color changes often reduce accuracy and consistency owing to human errors. To address this limitation, colorimetric sensors have been integrated with AI models. ML- and DL-based AI algorithms automate working protocols for color patterns such as color, shape, and size from image data, enabling analysis within seconds to hours. ML- and DL-based AI algorithms are being used to develop more sensitive, accurate, quick, automated, and reliable diagnostic tools. However, AI algorithms do not autonomously adapt and are pre-trained. This section provides an overview of ML and DL algorithms that enhance the performance of a colorimetric biosensor for virus detection.

### 3.1. ML-Based Colorimetric Detection of Viruses

The ML algorithm is a powerful approach for analyzing colorimetric signals and images obtained from viral assays. Conventional detection methods often have low accuracy because colorimetric outputs can be affected by ambient lighting conditions and background noise. ML algorithms address these challenges by analyzing complex color data and compensating for environmental variations. Recent viral detection platforms have successfully employed ML methods such as ANN, RF, and SVM.

#### 3.1.1. ANN for Virus Detection

**Figure 5:**
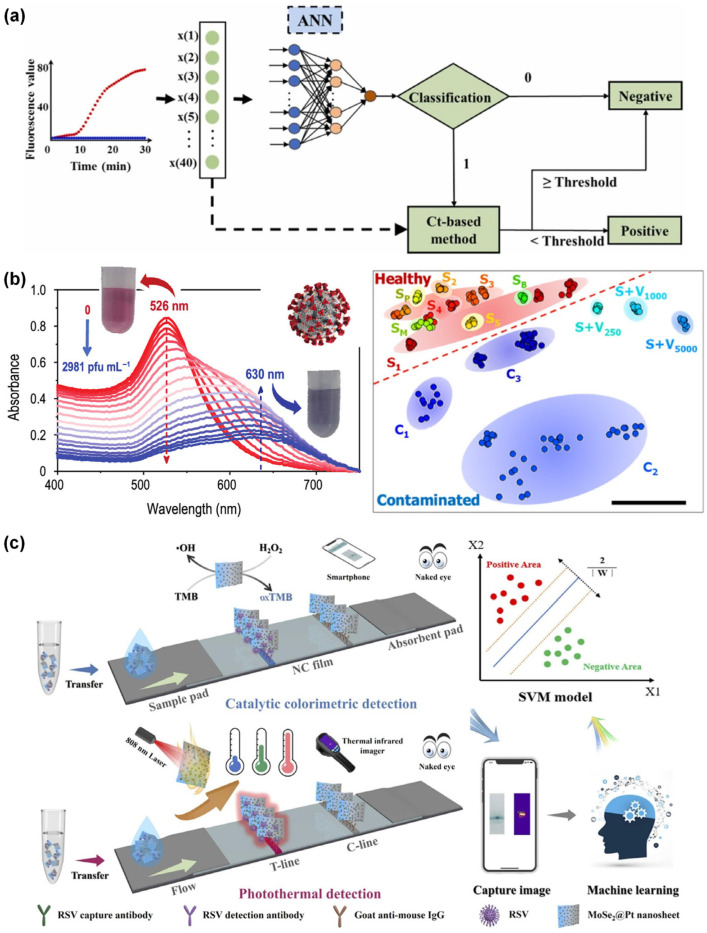
(**a**) Flowchart of a hybrid classification model based on the cycle threshold and ANN analysis (Ct+ANN) for virus identification; reprinted with permission from [37], copyright 2022 Elsevier. (**b**) Ultraviolet–visible spectra and photographic images of functionalized AuNPs solutions with RF model analysis of human saliva samples; reprinted with permission from [38], copyright 2022 ACS. (**c**) Schematic illustration of SVM-based catalytic colorimetric and photothermal detection using designed test strips; reprinted with permission from [40], copyright 2022 Elsevier.

Biosensor outputs are used by the ANN algorithm to learn nonlinear color and optical pattern signatures, enabling virus classification and quantification, even when signals are distorted by noise, variability, and light conditions. Miao et al. (2022) [37] reported a portable and real-time ANN-assisted capillary convection PCR system for detecting African swine fever virus (ASFV) in peripheral blood samples. A hybrid cycle threshold (Ct)-ANN analysis was designed to overcome the limitations of conventional Ct-PCR models in field environments. A polycarbonate capillary tube was used to heat the bottom, enabling thermal convection cycling for PCR amplification without the need for complicated instrumentation. Fluorescent signals generated during amplification were collected using a fiber-optics. A three-layer ANN model was employed to classify abnormal negative curves before applying the Ct-value-based decision logic. The Ct-ANN hybrid model achieved significantly higher detection accuracy (97.5%) compared with standard Ct analysis (47.5%). The Ct-ANN hybrid model successfully distinguished irregular negative curves from true positives, resulting in highly accurate ASFV detection in a low-cost handheld diagnostic system (Figure 5a) [37]. Overall, this demonstrates that integrating ANN with standard Ct analysis enables reliable ASFV detection under high-noise conditions (Table 3). Despite its improved performance, the Ct-ANN model remains limited by its reliance on PCR amplification and fluorescence color properties. Also, ANN-specific challenges include overfitting on small datasets, reliance on larger datasets, interpretability concerns, and high computational resource demand in resource-limited environments.

**Table 3:** Overview of ML and DL algorithms for colorimetric biosensor-based detection of viruses.

Sl. No.	Category/AI Algorithm	Target Viruses	Data Size	Accuracy	LOD/Linear Range	Reaction Time	Real Samples	Ref.
1	ML/ANN	ASFV	392	97.5%	5 × 10^2^ copies·mL^−1^/5 × 10^2^–5 × 10^6^ copies·mL^−1^	30 min	N.A.	[37]
2	ML/RF	SARS-CoV-2	1200	100%	0.28 PFUs·mL^−1^/7–2000 PFUs·mL^−1^	30 min	Saliva and river water	[38]
3	ML/SVM	SARS-CoV-2 and H1N1 influenza	500	95%	6.2 copies·mL^−1^/7.2–7.2 × 10^6^ copies·mL^−1^	13 min	Saliva	[39]
4	ML/SVM	H1N1, H3N2, FLUB, and SARS-CoV-2	200	97.5%	0.1 × 10^5^ copies·mL^−1^/0–20 × 10^5^ copies·mL^−1^	15 min	Nasal swabs	[40]
5	DL/CNN	SARS-CoV-2	595,339	98.4%	N.A./N.A.	15 min	Human blood	[41]
6	DL/CNN	SARS-CoV-2	726	99%	N.A./N.A.	N.A.	Nasal swabs	[42]
7	DL/CNN	SARS-CoV-2 and ASFV	2543	100%	58 total copies/0–7 × 10^5^ total copies	30 min	Human blood	[43]
8	DL/CNN	Human immunodeficiency virus	11,374	98.9%	N.A./N.A.	N.A.	Blood, serum, and plasma	[44]
9	DL/CNN	SARS-CoV-2	894	97.9%	N.A./N.A.	2 min	Naso-oropharyngeal swabs and saliva	[45]
10	DL/CNN	SARS-CoV-2 N-protein	2586	95.3%	0.074 ng/0.074–7.4 ng	15 min	N.A.	[46]
11	DL/CNN	SARS-CoV-2	1500	98%	0.156 ng·mL^−1^/N.A.	15 min	N.A.	[47]
12	DL/U-Net	SARS-CoV-2 N-protein	3146	96.5%	1 nM/1–100 nM	0.2 s	N.A.	[48]
13	DL/ResNet	SARS-CoV-2	213	100%	3.7 × 10^2^ copies·mL^−1^/N.A.	75 min	Nasopharyngeal swabs	[49]
14	DL/ResNet	SARS-CoV-2	N.A.	94.52%	2.5 pg·mL^−1^/0.1–10,000 pg·mL^−1^	15 min	Nasopharyngeal swabs	[50]
15	DL/ResNet	SARS-CoV-2	234	N.A.	160 ng·mL^−1^/625–10,000 pg·mL^−1^	20 min	Serum	[51]

Abbreviations: ASFV, African swine fever virus; FLUB, influenza B virus; PFU, plaque-forming unit; SARS-CoV-2, severe acute respiratory syndrome coronavirus-2.

#### 3.1.2. RF for Virus Detection

The RF algorithm is a robust ML model that uses decision trees to accurately perform classification and regression on nonlinear and multidimensional datasets. RF is highly effective for tasks such as gene expression profiling, spectral analysis, and smartphone-based color image identification. Materon et al. (2022) [38] employed RF to classify plasmonic colorimetric signals generated during SARS-CoV-2 detection using polyclonal antibody-functionalized AuNPs (f-AuNPs) and smartphone imaging. They developed a portable and instrument-free plasmonic biosensor that enables visual detection of SARS-CoV-2 in saliva and river water samples. This method relies on f-AuNPs undergoing virus-induced aggregation, which results in intense red-to-blue color changes that were readily captured using a smartphone. An RF classifier was trained on smartphone-extracted color features and ultraviolet–visible spectrum data from saliva samples. The RF model showed 100% accuracy in identifying SARS-CoV-2-positive and -negative samples within 30 min (Figure 5b) [38]. Overall, the results indicated that RF-based analysis of smartphone-captured colorimetric signals enables rapid and accurate detection of SARS-CoV-2 in real-world samples (Table 3), while ensemble learning with pruning improves robustness against overfitting and reduces model complexity.

#### 3.1.3. SVM for Virus Detection

The SVM algorithm identifies optimal separating hyperplanes in high-dimensional feature spaces, enabling the accurate detection of optical, thermal, and colorimetric patterns generated by biosensors. AbdElFatah et al. (2023) [39] implemented this approach to quantify nucleic acid biomarkers. The developed device (QolorEX) is a microfluidic diagnostic platform that provides enhanced reverse transcription-loop-mediated isothermal amplification (RT-LAMP) through plasmonic hot-electron injection, enabling rapid colorimetric detection of nucleic acids from viral and bacterial samples. Excitation of PSP–zinc oxide–aluminum plasmonic NP surfaces induces hot electron generation, resulting in up to a ninefold enhancement in nucleic acid polymerization and rapid phenol-red color shifts from pink to yellow. Colorimetric images of the microfluidic chamber were captured using a smartphone. The SVM classifier was integrated into the QolorEX system to analyze time-resolved RGB and HSV values in positive and negative RT-LAMP samples. Integration of the SVM model with the QolorEX platform enabled highly sensitive and selective detection of SARS-CoV-2, influenza A virus (H1N1), and bacteria with 95% accuracy and a total assay time of 13 min [39]. Xu et al. (2025) [40] developed molybdenum selenide (MoSe_2_) nanosheets decorated with PtNP (MoSe_2_@Pt) to form heterojunction nanozymes with enhanced catalytic and photothermal activities to fabricate dual-modal LFA strips for detecting respiratory syncytial virus (RSV), influenza A viruses, influenza B virus, and SARS-CoV-2. The MoSe_2_ nanosheets provided a high surface area with PtNPs loading, which enhanced peroxidase-like activity and heat-conversion efficiency. RSV binding to the test line generated an amplified blue color signal and photothermal responses. An SVM model integrated multimodal color and thermal data to enhance diagnostic accuracy. The SVM model enabled strong nonlinear identification, achieving an accuracy of 97.5% within a total assay time of 15 min (Figure 5c) [40]. Overall, combining SVM with advanced plasmonic- and nanozyme-based colorimetric and thermal biosensors enables quick, accurate, and sensitive virus detection. Therefore, this approach has strong potential for use in next-generation POCT diagnostics (Table 3). Both studies indicate that SVM can reliably classify time-dependent and high-dimensional POCT signals. The QolorEX platform reported by AbdElFatah et al. (2023) [39] enabled rapid DNA detection with 95% accuracy within 13 min. The MoSe_2_@Pt dual-mode LFA developed by Xu et al. (2025) [40] provided more robust multiplex virus sensing with high accuracy. However, SVM is computationally demanding and struggles with large and noisy datasets, which may limit real-time applications.

### 3.2. DL-Based Colorimetric Detection of Viruses

The DL algorithm substantially improves the accuracy of colorimetric virus detection by automatically learning complex visual patterns. However, DL algorithm does not autonomously adapt and is typically pre-trained. CNN models can effectively analyze a wide range of color intensities and complex variations in LFA images. U-Net- and ResNet-based frameworks have demonstrated outstanding performance in colorimetric detection to support real-time POCT for viral diagnostics.

#### 3.2.1. CNN for Virus Detection

**Figure 6:**
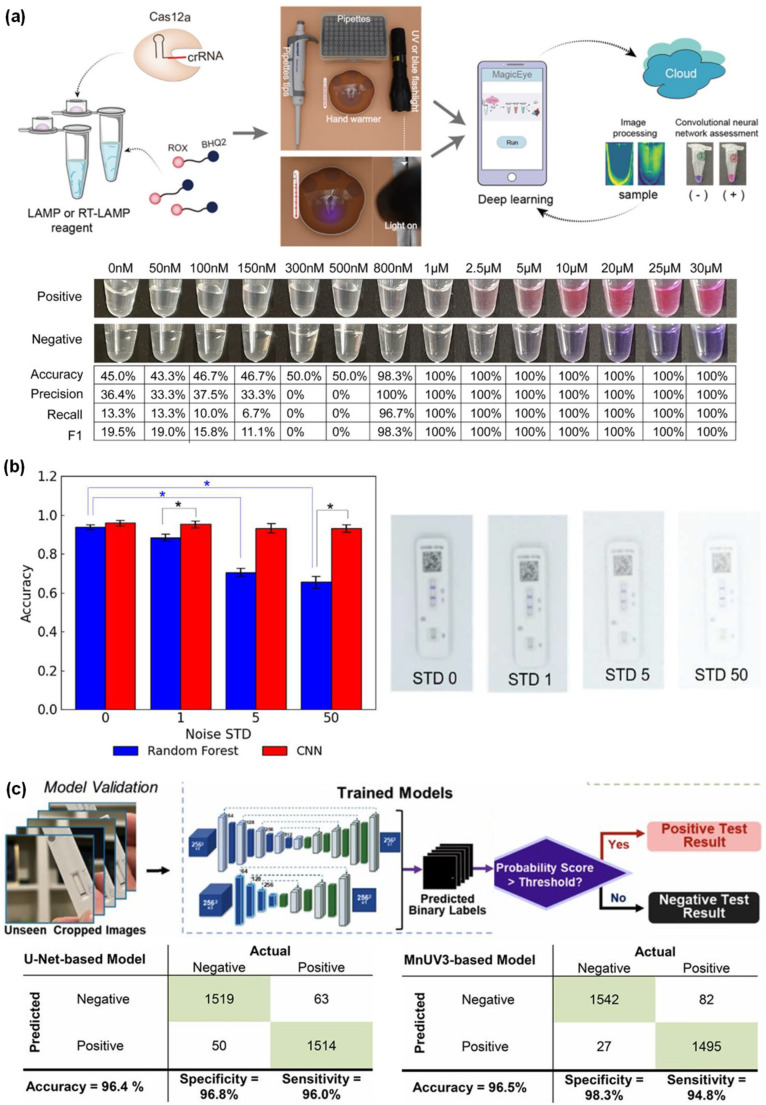
(**a**) Schematic diagram of naked-eye colorimetric detection of SARS-CoV-2 using a CNN-based model for binary classification under different deoxyribonucleic acid concentrations; reprinted with permission from [43], copyright 2022 ACS. (**b**) Classification accuracies of RF and CNN models trained on images with varying noise levels and the corresponding captured images; reprinted with permission from [46], copyright CC BY 4.0. (The asterisk indicates a statistically significant difference (*p* < 0.05)) and (**c**) Overview of the workflow for image preprocessing, model training, and model validation using universal-network (U-Net) and MobileNet U-Net Version-3 models for SARS-CoV-2 N detection and the respective results; reprinted with permission from [48], copyright CC BY 4.0.

The CNN algorithm automatically learns hierarchical visual features, making it highly effective for interpreting biosensor images and supporting clinical diagnostics. CNNs can extract deep spatial features enable accurate virus classification and identification even in the presence of complex data, noise, and manual errors. Wong et al. (2022) [41] reported a CNN that supported the visual auditing of home-based automated LFA test strips for detecting SARS-CoV-2 antibodies. The captured images varied significantly in lighting conditions and camera type. The CNNs were trained to detect test lines, invalid test strips, blood-leakage issues, and immunoglobulin G classifications such as negative, positive, and strongly positive. This CNN system was applied to 595,339 participants and achieved a scalable solution with 98.4% accuracy for national-level antibody surveillance [41]. Arumugam et al. (2023) [42] introduced a portable colorimetric biosensor for SARS-CoV-2 detection using smartphone imaging combined with DL. Analyte-induced reactions promote distinct color changes in paper-based biosensors. The CNN analyzed pixel-level spatial color features at different concentrations under uneven lighting and shadowing conditions. A deep CNN architecture extracted multiscale features from images to perform multiclass quantitative prediction 99–100% over 726 tests (350 positive, 376 negative) [42]. Xie et al. (2022) [43] developed a naked-eye colorimetric detection method for nucleic acids based on rapidly visually clustered regularly interspaced short palindromic repeats (RAVI-CRISPR) integrated with a CNN, which improved interpretation consistency under low line intensity and high background noise. A CNN with multiple convolutional layers was trained on thousands of LFA images for ROI extraction followed by positive/negative classification, and quantitative prediction. The RAVI-CRISPR system was 100% accuracy in detecting SARS-CoV-2 and ASFV in 2543 human blood samples (Figure 6a) [43]. Turbé et al. (2021) [44] conducted a large-scale real-world DL study on diagnostic LFA strips by collecting 11,374 field-acquired HIV rapid diagnostic test (RDT) images from rural South Africa. LFA strip images were captured by 60 field workers using a standard tablet device. The CNNs were trained to separate control and test lines within the ROI, even ambiguous lines that often cause human reading errors. The CNN architectures enabled scalable interpretation of RDTs for “REASSURED” diagnostics referring to real-time connectivity, ease of specimen collection, affordable, sensitivity, specificity, user-friendliness, rapidity, equipment-free operation, and deliverability [44]. Lee et al. (2024) [45] introduced “TIMESAVER” a hybrid CNN-long short-term memory DL architecture designed to significantly shorten assay time by 2 to 15 min while maintaining 97.9–99% accuracy. Initial color changes on the test line were recorded every few seconds. The DL model predicted positive outcomes before visible color development. Hybrid CNN models utilized to detect ROIs, extract spatial features, and analyze color evolution enabling SARS-CoV-2 detection within as short as 2 min in human naso-oropharyngeal swabs and saliva samples [45]. Davis and Tomitaka (2025) [46] introduced ML- and DL-based frameworks to convert smartphone-captured LFA images into quantitative analyte concentration readouts. The system employed lightweight ML and CNN models to quantify SARS-CoV-2 nucleocapsid (N) protein in a rapid antigen test rather than relying on subjective visual inspection. LFA strips containing different N-protein concentrations produced variable test-line intensities. Thousands of images were captured in a controlled imaging box with rotation, zoom, and brightness variations to mimic real-world smartphone conditions. The CNN learned the spatial intensity within the test window and achieved 95.3% accuracy with an LOD of 0.074 ng (Figure 6b) [46]. Lee et al. (2023) [47] developed an AI-integrated smartphone-based LFA platform for accurate SARS-CoV-2 antigen detection. Commercial LFA strips exhibited concentration-dependent color patterns on the test line. However, the environmental factors such as inconsistent lighting, camera angles, and shadows, reduced its accuracy. The system automatically detected LFA strips using YOLO and classified the results using a CNN. The platform employed YOLOv3 to identify test regions, a CNN for classification tasks, and data-automation techniques to enhance robustness. This approach achieved >98% accuracy demonstrating reliable weak-line interpretation across all smartphone models without requiring additional accessories. This method was evaluated 1500 samples, an LOD of 0.156 ng·mL^−1^, and assay time 15 min (Table 3) [47].

Across these studies, DL models based on CNNs can significantly improve smartphone- and LFA-based interpretation by learning robust features that overcome noise and are user-friendly. However, CNNs require large labeled datasets and computational power, making real-time deployment challenging in resource-limited areas. Wong et al. (2022) [41] demonstrate unmatched scalability for national surveillance by auditing strip validity across 595,339 users. Turbé et al. (2021) [44] showed strong real-world and filed-ready HIV quantitation under signal-to-noise conditions. In contrast, Lee et al. (2024) [45] achieved the fastest detection by predicting positive outcomes before visible color development using the CNN-LSTM model. Lee et al. (2023) [47] further improved robustness by integrating YOLO-based ROI detection with CNN classification across multiple smartphones models. The studies by Turbé et al. (2021) [44] and Lee et al. (2024) [45] were reported to lack key parameters such as LOD and concentration analysis, which limited overall robustness. Common challenges include light variation, ROI detection error, dataset sizes, and insufficient external validations. For further studies, Wong et al. (2022) [41], Turbé et al. (2021) [44], and Lee et al. (2024) [45] suggest that combined and multimodal DL approaches are essential for field readiness and large-scale reliable deployment in resource-limited devices.

#### 3.2.2. U-Net and ResNet for Virus Detection

U-Net is a DL algorithm specifically designed for precise localization and segmentation of important regions within images. U-Net enables colorimetric biosensor systems to focus exclusively on suitable color alterations by effectively segmenting the critical reaction zone. U-Net serves as a segmentation network that isolates key reaction regions, resulting in a precise focus on real color changes. Xue et al. (2025) [48] developed a DL platform based on U-Net integrated with CRISPR–Cas13 LFA strips for detecting the SARS-CoV-2 N protein. LFA images were captured with a smartphone under real-world lighting and background conditions. The system employs a U-Net segmentation network to automatically detect and isolate control lines from raw LFA photos without requiring manually controlled lighting. These U-Net segmentation outputs are processed by a secondary classifier to determine positive and negative results. U-Net exhibited strong robustness when applied to a dataset of 3146 smartphone images acquired with varying angles, lighting conditions, and backgrounds. The model achieved 96.5% detection accuracy within 0.2 s (Figure 6c) [48].

ResNet is a DL algorithm that maintains high accuracy while learning deep image features through residual connections. Its robust capability to capture complex color variations enables highly reliable classification of viral biosensor images. Jaroenram et al. (2022) [49] developed a dual-dye colorimetric RT-LAMP assay integrated with a RestNet-50-based image analysis tool for automated result interpretation. Images of the reaction tubes were captured using a smartphone camera. The ResNet-50 backbone extracted deep visual features such as hue, saturation, and chromatic transitions, which were passed into a detection transformer to classify the tubes and map each reaction to the corresponding sample location. This system achieved 100% accuracy with naked-eye and RT-LAMP interpretation, demonstrating highly stable color recognition under varying lighting conditions [49]. Zhao et al. (2025) [50] introduced an ultrasensitive SERS–LFA platform for SARS-CoV-2 N protein detection by combining Raman imaging with DL-based classification. ResNet-18 was employed as the classification network trained on the SERS imaging data for negative and positive signals based on different nanoprobe shapes. The ResNet-18 model achieved 94.52% accuracy with an LOD of 2.5 pg·mL^−1,^ approaching the theoretical Raman LOD. This model effectively suppresses false signals caused by membrane heterogeneity [50]. Tong et al. (2022) [51] developed an LFA platform using polydopamine NPs coupled with a smartphone-based RestNet-50 reader for quantitative neutralizing antibody detection. The LFA images were preprocessed and analyzed using the RestNet-50 feature extractor, which identifies band boundaries and generates a robust feature map. ResNet was trained to recognize intensity differences between the test and control lines, facilitating precise quantification. The system achieved an LOD of 160 ng_·_mL^−1^ and demonstrating highly accuracy across 50 clinical serum samples. ResNet-50 also ensured consistent performance across multiple smartphone models, thereby confirming its suitability for POCT (Table 3) [51].

Overall, Xue et al. (2025) [48] demonstrate excellent field robustness and automatically segment test and control lines from raw smartphone images within 0.2 s under variable light. Jaroenram et al. (2022) [49] and Tong et al. (2022) [51] showed that ResNet-based models provide stable tube and strip classification across different smartphones, while Zhao et al. (2025) [50] demonstrated their effective suppression of false signals arising from membrane heterogeneity. Collectively, U-Net and ResNet significantly enhance the precision, speed, and robustness of colorimetric and LFA-based viral diagnostics, enabling a highly accurate, smartphone-friendly, and lighting-independent solution. However, U-Net requires high-quality annotated datasets and may struggle with real-time segmentation under highly noisy conditions. ResNet faces challenges related to model interpretability and high computational requirements, which can limit clinical trust and deployment.

## 4. Conclusions and Challenges

This review provides a comparative overview of the rapid development of AI-assisted colorimetric biosensors as powerful tools for pathogen diagnostics. Conventional optical and molecular methods are time-consuming, prone to manual errors, and require sophisticated instrumentation. Conversely, colorimetric biosensors are simple, inexpensive, and compatible with POCT because they convert biorecognition events into visible color changes. AI integrated with colorimetric biosensors using ML and DL models can transform qualitative “yes/no” readouts into automated and quantitative platforms for detecting bacteria and viruses. In this review, we explored recent AI models for pathogen detection, including ANN, kNN, LDA, RF, SVM, CNN, YOLO, U-Net, and ResNet. These AI models have been effectively employed to analyze RGB, HSV, and spectral data derived from PCA, LFA, images, and spectra. AI approaches enable the multiplex identification of species, Gram-type classification, and AST of clinical and environmental samples. Similarly, viruses such as SARS-CoV-2, influenza, HIV, and hepatitis can be detected with high accuracy, a low LOD, and a short assay time using AI-assisted colorimetric biosensors. Importantly, AI improves the interpretation of weak test lines and mitigates issues caused by lighting variability, background noise, and complex data in LFA and POCT workflows.

However, several key concerns remain unresolved in current AI models. Many models are pre-trained on small, lower-diversity, controlled datasets (ML: 50 and DL: 213), and do not autonomously modify, which limits their robustness in real-world applications. The lack of standardized protocols for camera and smartphone usage, such as image acquisition, overfitting, data annotation, and performance reporting (for example, confusion matrix, precision, recall, F1-score, and ROC-AUC), makes model validation difficult. Batch-to-batch variation in sensing materials, matrix effects from complex samples, and data variability caused by differences in experimental setups and imaging acquisition (for example, camera model, length, lighting, white balance, background, and sensing protocol across laboratories) significantly limit reproducibility even when studying the same target. Moreover, limited model interpretability and insufficient calibration strategies pose significant challenges for regulatory approval and reliable deployment of the POCT system in resource-limited settings. To address these challenges, future research should prioritize developing large, diverse, and preferably open-access datasets to enhance the robustness and generalizability of AI-assisted biosensors. Incorporating real-world and multi-source data will be critical for improving system performance across varied environmental conditions.

## 5. Future Perspectives

In the near term, the development of lightweight and edge-ready AI architectures for fully on-site and POCT analysis devices is crucial for ensuring rapid, accurate, and cost-effective diagnostics. Additionally, XAI methods are essential for improving transparency, interpretability, and system trust in the clinical field. Most current AI-assisted biosensors rely on static models that do not autonomously extend beyond their training data. Addressing this limitation through online learning adaptation remains an important short-term objective. From the data perspective, ML models typically perform reliably with datasets of 100 to 5000 samples, whereas DL models require larger datasets (500 to 50,000) to achieve stable and accurate training. To ensure effective utilization of large datasets and fair comparison, the following key requirements should be fulfilled in order of increasing importance: data formatting and organization, data cleaning and noise removal, data normalization and scaling, label accuracy and consistency, dataset diversity and representativeness, reproducibility through proper documentation, data sufficiency size and balance, and independent validation. Future studies should standardize camera- and smartphone-based imaging acquisition, including camera model, length, lighting, white balance, and background, with common matrices of precision, recall, F1 score, and ROC-AUC. These measurements are critical for achieving robustness comparable to DL-based models.

Looking toward long-term goals, a major future vision is an autonomously trained and self-improving AI-driven biosensor capable of dynamically adapting sensing protocols and decision-making without pre-existing training data. Such a system would represent a significant advance beyond current AI platforms, enabling continuous learning and autonomous optimization in complex and evolving environments. To bridge the gap from laboratory research to real-world deployment, strategies must be established for development, validation, and implementation. Long-term future research directions include:

- **Standardization and validation:** Develop standardized protocols for image acquisition, data annotation, preprocessing, and performance reporting to ensure model reproducibility and universal applicability. The establishment of a centralized international committee involving regulatory agencies, institutions, and industry would facilitate harmonized guidelines and regulated clinical validation across laboratories worldwide.
- **Cause study-driven applications:** Practical implementations such as AI-powered LFA kits for infectious disease detection, food safety monitoring, and water quality monitoring can serve as examples for broad acceptance.
- **Collaborative research:** Strategic partnerships with hospitals, research institutes, and global health organizations will support the creation of diverse datasets and adaptable AI models.
- **Proof-of-concept projects:** Deployment of proof-of-concept studies to integrate AI-enhanced biosensors in clinical, environmental, and industrial areas, such as POCT and multi-sensor devices in public health.
- **Infrastructure integration:** Incorporate IoT-enabled and AI-driven biosensors into a large-scale health and environmental surveillance system to support real-time diagnostics and global monitoring.
- **Multimodal sensor integration:** Integrating colorimetric sensing with complementary models such as acoustic, electrochemical, viscosity, and thermal modalities can enhance diagnostic accuracy and response speed. The application of XAI tools such as SHAP, LIME, and Grad-CAM will further enhance interpretability and acceptance. In the long term, unified multimodal AI frameworks capable of jointly analyzing heterogeneous sensor data are expected to significantly improve pathogen detection in multiple environments.

Overall, addressing these near-term and long-term directions will help transform AI-assisted colorimetric biosensors from proof-of-concept demonstrations to practical, scalable, trustworthy, and multimodal diagnostic tools capable of addressing current bottlenecks and future global health challenges.

## Figures and Tables

**Table 1:** Classification of AI-assisted colorimetric biosensors.

Category	AI Algorithm	Role in Colorimetric Sensor	Working Principle	Learning Type/Input Data Type	Application Conditions	Data Requirements	Practical Considerations
Machine learning (ML)	Support vector machine (SVM)	Classifies of color-intensity features (+ or −)	Constructs a separating hyperplane color space	Supervised/handcrafted images	Good for high-dimensional and lower data	Labeled and clear classes data	Requires careful tuning
	Random forest (RF)	Predicts concentrations from RGB/HSV	Builds multiple decision trees to enhance accuracy	Supervised/handcrafted images	Works for classification and regression	Labeled data and mixed features	Less sensitive and may fail to detect computational outliers
	k-Nearest neighbor (kNN)	Identification of unknown nearest color	Classifies by comparing its color vector with kNN	Supervised/handcrafted images	Best for small and well-clustered datasets	Larger labeled datasets for nearest color	Slow dataset growth and scale-sensitive
	Linear discriminant analysis (LDA)	Classification of analyte categories using color	Linear projection maximizing class separation	Supervised/handcrafted images	Best for simple and clear class separability	Labeled datasets for normal distribution	Weak linear patterns
	Artificial neural network (ANN)	Mapping of color intensity	Models nonlinear relationships and out mapping	Supervised/handcrafted image	Complex and non-linear data	Labeled feature engineering	Overfitting and high time for training
Deep learning (DL)	Convolutional neural network (CNN)	Extracts spatial and texture color	Convolution and pooling layers feature	Supervised/raw images	Strong for image-based recognition	Larger labeled datasets	High graphics processing unit acceleration
	You only look once (YOLO)	Identification of control and positive line	One stage detector with boxes and classes	Supervised/raw images	Real-time detection	Image with bounding boxes and labels	Efficient but may miss crowded data
	U-shaped network (U-Net)	Segment ROI/reactive zone for quantification	Encoder−decoder network for pixel-wise	Supervised/raw images	Excellent for segmentation	Datasets with pixel-level masks	Effective for high computed cost
	Residual network (ResNet)	Deep transfer learning for multi-analyte	Residual blocks enable very deep image	Supervised/raw images	Great for deep feature large datasets	Larger labeled datasets	Highly efficient but expensive
	MobileNet	On-site color analysis	Depth-wise separable color analysis	Supervised/raw images	Ideal for mobile	Larger labeled images	Speed but low accuracy

## Data Availability

Data are contained within the article.
